# A Drop of Water

**Published:** 1889-12

**Authors:** Herbert Spencer


### A DROP OF WATER.

"Think you that a drop of water, which to the vulgar eye is but a drop of water, loses everything in the eye of the physicist, who knows that its elements are held together by a force which, if suddenly liberated, would produce a flash of lightning? Think you that what is carelessly looked upon by the uninitiated as a mere snow flake does not suggest higher associations to one who has seen through a microscope the wondrously varied and elegant forms of snow crystals? Think you that the rounded rock, marked with parallel scratches, calls up as much poetry in an ignorant mind as in the mind of a geologist, who knows that on this rock a glacier slid a million years ago? The truth is, that those who have never entered upon scientific pursuits are blind to most of the poetry by which they are surrounded. Whoever has not in youth collected plants and insects knows not half the halo of interest which lanes and hedgerows can assume. Whoever has not sought for fossils has little idea of the poetical associations that surround the places where imbedded treasures are found. Whoever at the seaside has not had a microscope and aquarium has yet to learn what the highest pleasures of the seaside are."—*Herbert Spencer.*

